# Epigenetic hypomethylation and upregulation of matrix metalloproteinase 9 in Kawasaki disease

**DOI:** 10.18632/oncotarget.19650

**Published:** 2017-07-28

**Authors:** Ho-Chang Kuo, Sung-Chou Li, Lien-Hung Huang, Ying-Hsien Huang

**Affiliations:** ^1^ Department of Pediatrics, Kaohsiung Chang Gung Memorial Hospital, Chang Gung University College of Medicine, Kaohsiung, Taiwan; ^2^ Kawasaki Disease Center, Kaohsiung Chang Gung Memorial Hospital, Kaohsiung, Taiwan; ^3^ Department of Medical Research, Genomics and Proteomics Core Laboratory, Kaohsiung Chang Gung Memorial Hospital, Chang Gung University College of Medicine, Kaohsiung, Taiwan; ^4^ Department of Pediatrics, Chiayi Chang Gung Memorial Hospital, Kaohsiung, Taiwan

**Keywords:** matrix metalloproteinase, genetic methylation, Kawasaki disease, Immunology and Microbiology Section, Immune response, Immunity

## Abstract

**Background:**

Kawasaki disease (KD) is a type of febrile coronary vasculitis occurring in children. Some researchers have suggested that changes in genetic signatures, such as matrix metalloproteinases (MMPs), are critical markers for cardiovascular diseases. This study aims to provide a comprehensive survey of global DNA methylation levels and MMP transcripts of KD patients compared to control subjects.

**Materials and Methods:**

For chips studies, we recruited a total of 18 KD patients, prior to receiving intravenous immunoglobulin (IVIG) and at least 3 weeks after IVIG treatment, as well as 18 healthy and 18 febrile control subjects. We applied Illumina HumanMethylation450 BeadChip and Affymetrix GeneChip® Human Transcriptome Array 2.0 to evaluate their CpG markers and expression levels, respectively. Then we used a separate cohort to carry out real-time quantitative PCR validations of mRNA levels.

**Results:**

The expressions of mRNA levels of MMP-8, -9, and -25 were significantly upregulated in KD patients compared to the healthy and febrile controls. Once KD patients underwent IVIG treatment, these MMPs considerably decreased. In particular, the methylation status of CpG sites of MMP-9 indicated a significant opposite tendency between both stages of not only the KD samples but also the controls. We also observed the mRNA level of MMP-9 to be higher in KD patients with coronary arterial lesion formation.

**Conclusion:**

This study is the first to report epigenetic hypomethylation, an increased MMP-9 transcript, and the upregulation of MMP-9 in KD patients who had formed coronary arterial lesions.

## INTRODUCTION

Kawasaki disease (KD), also known as mucocutaneous lymph node syndrome or infantile periarteritis nodosa, inflames the walls of both small- and medium-sized blood vessels (vasculitis), particularly coronary arteries, throughout the body. KD typically affects children under the age of five years [1]. The most serious cardiovascular complications of KD are the result of coronary artery lesions (CALs) caused by inflammation, including myocardial infarctions, coronary artery fistula formations [2], coronary artery dilatations, and coronary artery aneurysms (CAAs) [3]. Although this disease can be controlled, nearly 20% of children who do not receive treatment suffer a CAA [2], which may lead to death in severe cases.

Matrix metalloproteinases (MMPs) may maintain the structure and function of coronary vascular walls [4]. MMP-9 and -7 are well known to influence CAA formation in KD [5, 6]. Shimizu *et al.* reported that haplotype analyses showed MMP-3 and -12 in the gene cluster on Chromosome 11 in CAA formation in KD patients [7]. The proinflammatory cytokine tumor necrosis factor-α (TNF-α) that stimulates MMP-9 production was also correlated with vascular damage in a KD model [8].

Epigenetics describes the acetylation pattern of the genome and DNA methylation and subsequently leads to changes in the chromatin structure [9]. In short, DNA methylation can deactivate genes through DNA methyltransferases [10]. In contrast, demethylation alterations of CpG sites indicate a contradictory change in gene expression [11]. In previous studies, we found that IVIG treatment significantly altered methylation patterns in KD patients [12] and that KD patients demonstrated considerably increased mRNA expression in TLRs and hypomethylation at the gene promoters of TLRs [13]. IVIG treatment can restore the methylation level of TLRs and decrease these TLRs’ mRNA expression [13]. No studies have yet surveyed all the MMP-1∼28 in the same report or performed analysis with methylation profiles. Therefore, in this study, we comprehensively examined the mRNA expressions of MMPs-1∼28 and analyzed changes in methylation levels in two different stages of KD patients, as well as two types of controls.

## RESULTS

### Differential expression of MMP mRNA levels among KD patients and controls and changes after IVIG treatment

**Figure 1:**
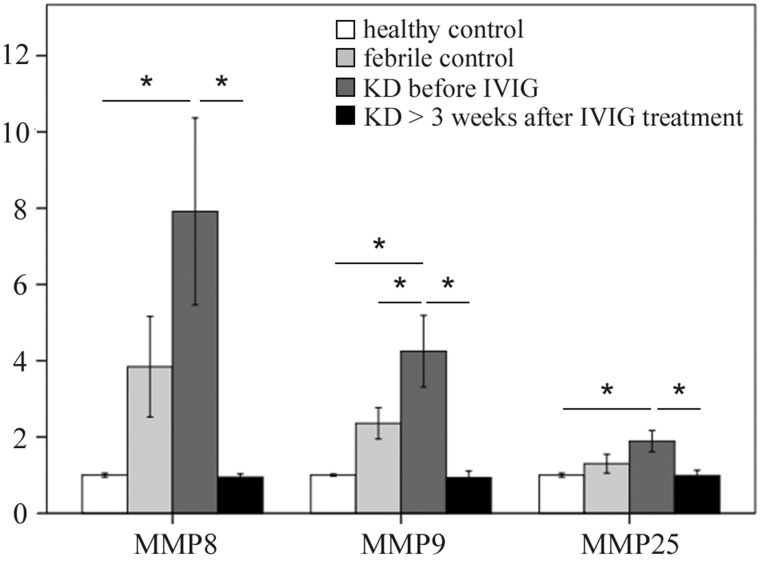
Comparison of matrix metalloproteinase (MMP) -8, -9, and -25 mRNA expressions by GeneChip^®^ Human Transcriptome Array 2.0 between acute-stage Kawasaki disease patients and control subjects * indicates significance (*p* < 0.05). Data are expressed as mean ± standard error for the three replications.

To investigate MMP1-28 transcripts expression, we used Affymetrix GeneChip^®^ Human Transcriptome Array 2.0 to determine their expression levels. Table 1 shows that KD patients had a differential expression of MMPs when compared to both the healthy and febrile control subjects. The mRNA levels of MMP-8, -9, and -25 were significantly higher in KD patients than in the healthy control and febrile control groups. These values significantly decreased in KD patients after undergoing IVIG treatment (Figure 1). We observed no noteworthy differences in the remaining MMPs among the groups or in KD patients following IVIG treatment.

**Table 1:** Transcripts expressions of matrix metalloproteinases (MMPs) between Kawasaki disease patients and control subjects

Symbol	RefSeq	Fold-Change	*p* value	Fold-Change	*p* value	Fold-Change	*p* value
		(KD1 *vs*. HC)	(KD1 *vs*. HC)	(KD1 *vs*. FC)	(KD1 *vs*. FC)	(KD3 *vs*. KD1)	(KD3 *vs*. KD1)
MMP1	NM_001145938	1.067	0.267	-1.046	0.425	-1.053	0.366
MMP2	NM_001127891	-1.004	0.970	-1.005	0.960	1.092	0.363
MMP3	NM_002422	1.009	0.865	-1.085	0.163	1.014	0.803
MMP7	NM_002423	1.011	0.876	-1.038	0.582	-1.001	0.990
MMP8	NM_002424	7.143	0.001*	2.176	0.083	-7.587	0.001*
MMP9	NM_004994	4.062	0.000*	1.771	0.037*	-4.481	0.000*
MMP10	NM_002425	-1.008	0.888	-1.104	0.093	1.020	0.709
MMP11	NM_005940	-1.024	0.825	-1.042	0.709	1.097	0.407
MMP12	NM_002426	1.050	0.389	-1.046	0.427	-1.013	0.814
MMP13	NM_002427	1.008	0.859	-1.100	0.060	1.029	0.531
MMP14	NM_004995	1.072	0.449	-1.059	0.531	1.029	0.756
MMP15	NM_002428	-1.023	0.817	-1.056	0.588	1.064	0.537
MMP16	NM_005941	1.007	0.928	-1.115	0.171	1.049	0.528
MMP17	NM_016155	-1.024	0.797	-1.086	0.388	1.035	0.717
MMP19	NM_002429	1.055	0.580	-1.017	0.864	-1.001	0.988
MMP20	NM_004771	-1.014	0.789	-1.093	0.111	1.026	0.620
MMP21	NM_147191	-1.036	0.546	-1.034	0.567	1.059	0.336
MMP23B	NM_006983	-1.060	0.580	-1.070	0.527	1.084	0.449
MMP24	NM_006690	-1.014	0.904	-1.027	0.822	1.052	0.669
MMP25	NM_022468	1.859	0.013*	1.473	0.082	-1.915	0.010*
MMP26	NM_021801	-1.030	0.619	-1.093	0.157	1.069	0.272
MMP27	NM_022122	1.000	0.999	-1.047	0.438	1.055	0.369
MMP28	NM_001032278	1.011	0.914	-1.015	0.877	1.029	0.767

KD1: Kawasaki disease before IVIG treatment; KD3: Kawasaki disease > 3 weeks after IVIG treatment; FC: febrile control; HC: healthy control.

### Significantly altered CpG sites on MMPs between KD patients and controls

**Figure 2:**
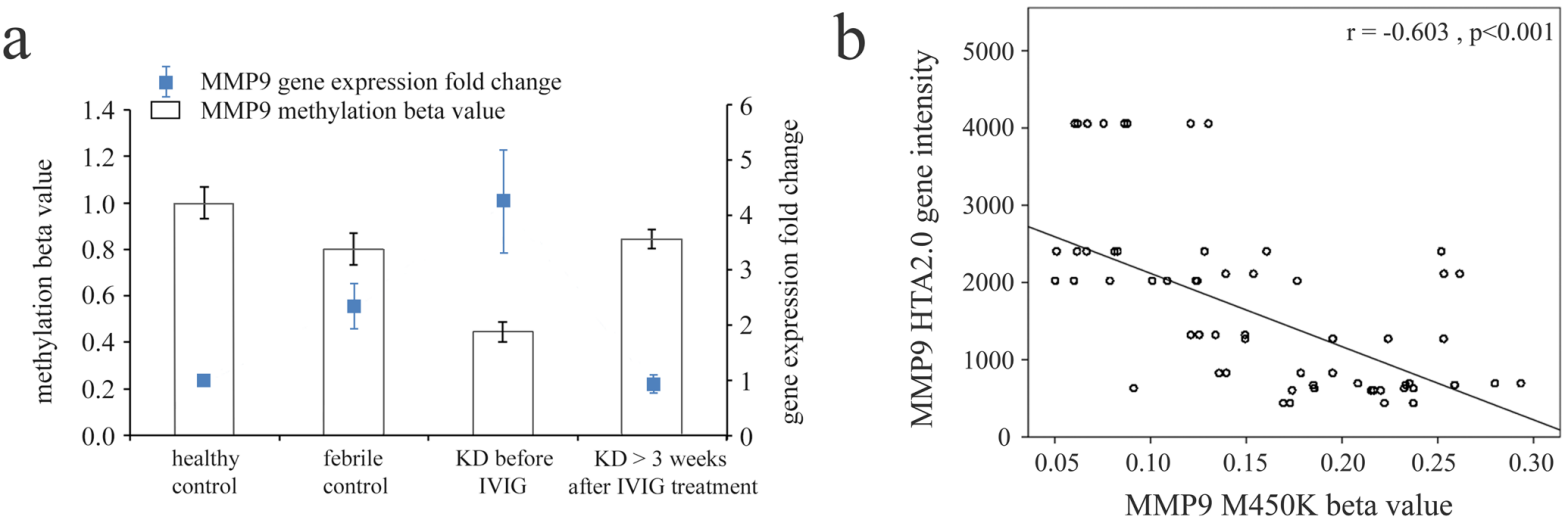
Integration of CpG marker (cg10505873) methylation pattern and gene expression profile of matrix metalloproteinase (MMP)-9 **a**. The methylation patterns of the representative CpG marker and gene expression profile of MMP-9 showed negative tendencies and were observed to change in both the healthy and febrile control subjects, as well as KD patients before and after receiving intravenous immunoglobulin treatment. The histogram and curve are presented as mean ± standard error. **b**. We used scatter plots to demonstrate the correlations between mRNA levels and DNA methylation, which show that mRNA levels were negatively correlated with DNA methylation (Pearson’s correlation coefficient approximately -0.603 and *p* < 0.001).

We adopted Illumina HumanMethylation450 BeadChip (Illumina) to assess the methylation patterns of CpG sites on MMPs between KD patients and both types of control subjects (Table 2a-2g). The MMP methylation levels were found to vary considerably in the acute stage of KD patients compared to the healthy and febrile controls (Table 2a-2g). Furthermore, the methylation levels of MMP1-28 were significantly lower in acute-stage KD patients compared to the healthy and febrile controls (Table 2a-2g). After KD patients underwent IVIG treatment, the methylation status of MMP-2, -9, -14, -15, and -16 was significantly increased, while the remaining MMPs were significantly decreased (Table 2a-2g). Decreased methylation results in greater gene expression [12], so we focused on the correlation between DNA methylation patterns and gene expressions. As shown in Figure 2, MMP-9 demonstrates a hypo-methylated status in KD patients not yet treated with IVIG compared to the control subjects and the KD patients already treated with IVIG. Therefore, the mRNA expression level and DNA methylation of MMP-9 represent a negative correlation, thus indicating that DNA methylation can repress gene expression.

**Table 2a:** Methylation patterns of CpG sites on matrix metallo proteinase between Kawasaki disease patients and control subjects

Target ID	Symbol	Fold-Change	*p*-value	Fold-Change	*p*-value	Fold-Change	*p*-value
		(KD1 *vs*. HC)	(KD1 *vs*. HC)	(KD1 *vs*. FC)	(KD1 *vs*. FC)	(KD3 *vs*. KD1)	(KD3 *vs*. KD1)
cg02212280	MMP1	-1.005	0.086	1.010	0.003*	-1.046	0.000*
cg11531628		-1.001	0.652	-1.003	0.206	1.005	0.029*
cg13759446		-1.001	0.002*	1.005	0.089	-1.017	0.000*
cg14543953		-1.003	0.629	1.004	0.529	-1.070	0.000*
cg16575408		-1.002	0.488	1.002	0.408	-1.014	0.000*
cg18733315		-1.012	0.000*	-1.001	0.708	-1.003	0.389
cg00078334	MMP2	-1.022	0.000*	-1.018	0.000*	1.019	0.000*
cg00087906		1.001	0.463	-1.001	0.276	1.005	0.000*
cg01821058		-1.003	0.071	-1.004	0.041*	1.007	0.000*
cg02458945		-1.003	0.028*	1.004	0.004*	-1.003	0.009*
cg07582157		-1.010	0.003*	-1.010	0.003*	1.041	0.000*
cg08318842		-1.010	0.000*	-1.010	0.000*	1.015	0.000*
cg09530163		-1.009	0.003*	-1.009	0.002*	1.002	0.520
cg10079740		-1.003	0.015*	1.003	0.012*	-1.001	0.238
cg12317456		-1.011	0.000*	-1.007	0.012*	1.004	0.129
cg14400118		-1.007	0.012*	-1.014	0.000*	1.006	0.047*
cg22950163		-1.010	0.000*	-1.015	0.000*	1.013	0.000*
cg25029998		-1.011	0.263	-1.003	0.793	-1.008	0.408
cg25349472		-1.004	0.715	1.002	0.850	1.003	0.780
cg26795346		-1.007	0.024*	1.002	0.568	-1.018	0.000*
cg27016988		-1.010	0.016*	-1.001	0.893	1.011	0.011*
cg27279620		-1.004	0.371	-1.016	0.000*	1.024	0.000*
cg27642062		-1.001	0.433	-1.005	0.004*	1.006	0.003*
cg01027553	MMP3	-1.007	0.089	-1.007	0.120	1.006	0.133
cg03168922		-1.008	0.185	-1.002	0.786	-1.015	0.009*
cg03231596		-1.019	0.008*	-1.009	0.209	1.009	0.215
cg16466334		-1.009	0.017*	-1.010	0.007*	-1.014	0.000*
cg17145397		-1.010	0.015*	1.002	0.613	-1.012	0.007*
cg18113270		-1.003	0.319	1.000	0.875	-1.016	0.000*
cg01813071	MMP7	-1.023	0.000*	-1.009	0.098	1.053	0.000*
cg04059146		-1.010	0.020*	1.002	0.650	-1.045	0.000*
cg17707274		-1.047	0.000*	-1.043	0.000*	1.096	0.000*
cg20645973		-1.028	0.000*	-1.013	0.011*	-1.007	0.140
cg24963041		-1.002	0.516	1.007	0.063	-1.025	0.000*
cg25511807		-1.137	0.000*	-1.064	0.001*	1.053	0.005*
cg01092036	MMP8	-1.011	0.000*	1.004	0.180	-1.015	0.000*
cg00182727	MMP9	-1.025	0.000*	-1.026	0.000*	1.017	0.000*
cg00732815		-1.047	0.000*	-1.031	0.000*	1.046	0.000*
cg02310296		-1.044	0.000*	-1.029	0.000*	1.025	0.000*
cg08176368		1.004	0.127	1.010	0.001*	-1.024	0.000*

**Table 2b:** Methylation patterns of CpG sites on matrix metallo proteinase between Kawasaki disease patients and control subjects

Target ID	Symbol	Fold-Change	*p*-value	Fold-Change	*p*-value	Fold-Change	*p*-value
		(KD1 *vs*. HC)	(KD1 *vs*. HC)	(KD1 *vs*. FC)	(KD1 *vs*. FC)	(KD3 *vs*. KD1)	(KD3 *vs*. KD1)
cg10505873	MMP9	-1.091	0.000*	-1.057	0.000*	1.064	0.000*
cg20925811		-1.049	0.000*	-1.050	0.000*	1.025	0.039*
cg21451869		-1.024	0.000*	-1.029	0.000*	1.020	0.001*
cg23353432		-1.006	0.022*	-1.011	0.000*	1.012	0.000*
cg26132320		-1.019	0.025*	-1.008	0.295	-1.030	0.000*
cg27372994		-1.023	0.003*	-1.011	0.159	1.007	0.385
cg15259031	MMP10	-1.011	0.006*	-1.004	0.294	-1.004	0.332
cg24960563		-1.001	0.490	-1.001	0.314	1.002	0.189
cg00771653	MMP11	-1.019	0.002*	1.002	0.744	-1.046	0.000*
cg03207310		-1.005	0.009*	1.000	0.798	1.004	0.032*
cg03290040		-1.005	0.023*	-1.008	0.000*	1.019	0.000*
cg04058144		-1.001	0.335	-1.003	0.054	1.004	0.002*
cg08141518		-1.026	0.147	1.000	0.993	-1.045	0.015*
cg09133003		-1.005	0.476	-1.002	0.779	-1.023	0.004*
cg12256538		-1.014	0.000*	-1.013	0.000*	1.037	0.000*
cg12395479		-1.008	0.033*	-1.014	0.000*	1.003	0.372
cg13361393		1.013	0.002*	1.017	0.000*	-1.029	0.000*
cg15808568		-1.007	0.759	1.041	0.065	-1.018	0.412
cg16159491		-1.002	0.228	-1.004	0.044 *	1.010	0.000*
cg18240463		1.003	0.695	-1.002	0.771	-1.015	0.025*
cg19934709		-1.002	0.419	1.011	0.000*	-1.012	0.000*
cg20202552		-1.009	0.000*	-1.000	0.973	-1.007	0.002*
cg21776003		1.007	0.009*	-1.002	0.452	-1.006	0.029*
cg23261640		1.008	0.260	1.021	0.004*	-1.002	0.763
cg23327483		1.004	0.028*	1.010	0.000*	-1.006	0.001*
cg27532722		-1.030	0.000*	-1.009	0.173	1.031	0.000*
cg02368591	MMP12	-1.015	0.021*	1.011	0.070	-1.031	0.000*
cg09002726		-1.020	0.001*	1.004	0.503	-1.014	0.017*
cg14948108		-1.008	0.038*	1.007	0.078	-1.023	0.000*
cg19304432		-1.005	0.128	1.006	0.060	-1.022	0.000*
cg20487452		-1.019	0.012*	1.001	0.925	-1.020	0.008*
cg23979520		-1.010	0.183	1.002	0.775	-1.039	0.000*
cg03067994	MMP13	-1.009	0.005*	1.001	0.689	-1.037	0.000*
cg10085326		-1.007	0.439	1.005	0.526	-1.067	0.000*
cg13041032		-1.001	0.663	1.006	0.004*	-1.027	0.000*
cg14995062		-1.007	0.216	1.009	0.086	-1.032	0.000*
cg16919569		-1.018	0.028*	-1.008	0.293	-1.000	0.997
cg19620758		1.012	0.203	1.019	0.045*	-1.049	0.000*
cg22658979		-1.006	0.042*	-1.001	0.792	-1.007	0.010*
cg24301681		-1.016	0.001*	-1.009	0.031*	-1.018	0.000*

**Table 2c:** Methylation patterns of CpG sites on matrix metallo proteinase between Kawasaki disease patients and control subjects

Target ID	Symbol	Fold-Change	*p*-value	Fold-Change	*p*-value	Fold-Change	*p*-value
		(KD1 *vs*. HC)	(KD1 *vs*. HC)	(KD1 *vs*. FC)	(KD1 *vs*. FC)	(KD3 *vs*. KD1)	(KD3 *vs*. KD1)
cg00691240	MMP14	-1.009	0.571	1.012	0.421	-1.038	0.015*
cg01016847		-1.051	0.000*	-1.026	0.001*	1.035	0.000*
cg01508380		-1.078	0.000*	-1.049	0.002*	1.027	0.070
cg04556361		-1.001	0.885	1.012	0.009*	-1.045	0.000*
cg05931439		-1.061	0.000*	-1.033	0.005*	1.024	0.042*
cg08321366		-1.077	0.000*	-1.059	0.000*	1.023	0.039*
cg09208010		-1.140	0.000*	-1.085	0.000*	1.070	0.001*
cg10418289		-1.081	0.000*	-1.057	0.000*	1.090	0.000*
cg10599444		-1.067	0.000*	-1.044	0.000*	1.037	0.001*
cg13094752		-1.085	0.000*	-1.049	0.001*	1.007	0.594
cg16119835		-1.019	0.000*	-1.012	0.020*	1.014	0.005*
cg18525873		-1.004	0.436	1.005	0.331	1.002	0.638
cg25594542		-1.015	0.000*	-1.003	0.384	-1.012	0.000*
cg27020028		-1.006	0.078	1.006	0.123	-1.008	0.030*
cg00247629	MMP15	-1.009	0.127	-1.001	0.887	-1.008	0.170
cg00963305		1.002	0.095	-1.004	0.000*	1.004	0.000*
cg01812337		-1.007	0.348	-1.011	0.147	1.056	0.000*
cg04211309		-1.016	0.000*	-1.002	0.491	-1.013	0.000*
cg05910755		-1.000	0.963	1.009	0.000*	-1.005	0.000*
cg06649282		-1.022	0.000*	-1.010	0.021*	-1.022	0.000*
cg06856889		-1.008	0.003*	-1.000	0.878	1.006	0.026*
cg08082374		-1.006	0.193	1.016	0.001*	1.028	0.000*
cg08514765		-1.011	0.000*	-1.005	0.017*	-1.000	0.927
cg08552042		-1.007	0.068	1.013	0.001*	-1.000	0.945
cg08877948		-1.005	0.215	-1.009	0.037*	1.026	0.000*
cg08943809		1.003	0.214	1.016	0.000*	-1.017	0.000*
cg10396713		-1.043	0.001*	-1.022	0.085	1.004	0.747
cg16181803		1.000	0.955	-1.009	0.000*	1.006	0.001*
cg16652241		1.001	0.616	-1.005	0.018*	1.008	0.000*
cg18333626		-1.001	0.793	-1.004	0.451	1.011	0.040*
cg18442019		-1.009	0.041*	-1.006	0.141	1.007	0.100
cg20566643		-1.006	0.001*	-1.010	0.000*	1.013	0.000*
cg20751926		-1.020	0.013*	-1.022	0.008*	-1.005	0.524
cg24306779		-1.001	0.811	-1.011	0.000*	1.004	0.154
cg25449950		1.015	0.095	-1.002	0.856	1.004	0.657
cg26725183		-1.003	0.335	1.017	0.000*	1.011	0.001*
cg26919014		-1.018	0.000*	-1.004	0.225	-1.004	0.233
cg27208052		-1.033	0.000*	-1.006	0.448	1.019	0.017*
cg00021786	MMP16	-1.009	0.169	-1.008	0.210	1.011	0.105
cg02889488		1.001	0.796	1.026	0.000*	-1.018	0.000*

**Table 2d:** Methylation patterns of CpG sites on matrix metallo proteinase between Kawasaki disease patients and control subjects

Target ID	Symbol	Fold-Change	*p*-value	Fold-Change	*p*-value	Fold-Change	*p*-value
		(KD1 *vs*. HC)	(KD1 *vs*. HC)	(KD1 *vs*. FC)	(KD1 *vs*. FC)	(KD3 *vs*. KD1)	(KD3 *vs*. KD1)
cg03210866	MMP16	-1.019	0.277	1.013	0.437	-1.030	0.090
cg04684553		1.016	0.018*	1.008	0.205	1.070	0.000*
cg05033271		1.006	0.383	1.000	0.981	1.107	0.000*
cg07609388		-1.006	0.029*	-1.026	0.000*	1.019	0.000*
cg08624180		-1.007	0.050*	-1.011	0.003*	1.018	0.000*
cg11536457		-1.001	0.685	1.000	0.935	1.025	0.000*
cg14644752		1.001	0.781	1.015	0.000*	1.033	0.000*
cg16852892		-1.007	0.005*	-1.006	0.032*	1.010	0.000*
cg22405982		-1.004	0.078	1.002	0.460	-1.001	0.470
cg24403959		-1.003	0.544	-1.010	0.082	-1.002	0.763
cg24456365		-1.006	0.112	-1.004	0.266	-1.011	0.008*
cg26075830		-1.021	0.000*	-1.008	0.059	1.003	0.442
cg02309230	MMP17	1.016	0.109	1.032	0.002*	1.003	0.756
cg02714882		-1.002	0.097	1.002	0.055	-1.002	0.065
cg03064145		-1.004	0.048*	-1.011	0.000*	1.007	0.003*
cg04346459		-1.036	0.045*	-1.002	0.907	1.028	0.114
cg07699454		-1.022	0.016*	1.001	0.863	1.016	0.067
cg10105149		-1.008	0.037*	-1.007	0.065	-1.004	0.308
cg10673839		1.003	0.487	1.003	0.468	-1.015	0.000*
cg11510557		1.000	0.720	1.009	0.000*	-1.010	0.000*
cg13082201		-1.002	0.229	-1.006	0.002*	1.009	0.000*
cg14215776		1.001	0.131	-1.003	0.000*	1.006	0.000*
cg15454599		-1.006	0.004*	-1.006	0.002*	1.006	0.005*
cg20393882		-1.007	0.248	1.009	0.147	-1.000	0.981
cg20844545		-1.000	0.838	-1.005	0.001*	1.004	0.009*
cg23477406		-1.001	0.563	-1.004	0.000*	1.004	0.000*
cg24493940		-1.003	0.550	1.000	0.930	-1.021	0.000*
cg25531700		-1.004	0.060	-1.009	0.000*	1.014	0.000*
cg26326372		-1.021	0.153	-1.032	0.031*	1.020	0.172
cg26356412		-1.019	0.002*	-1.016	0.009*	1.023	0.000*
cg26569074		-1.009	0.001*	-1.017	0.000*	-1.004	0.133
cg27207041		1.001	0.657	1.006	0.005*	-1.002	0.391
cg27305698		-1.006	0.045*	1.002	0.559	-1.008	0.006*
cg03509949	MMP19	-1.160	0.000*	-1.096	0.000*	1.126	0.000*
cg09907201		-1.023	0.001*	-1.018	0.008*	-1.029	0.000*
cg16936370		-1.021	0.015*	-1.019	0.022*	1.030	0.001*
cg17865265		-1.124	0.000*	-1.068	0.000*	1.074	0.000*
cg18787783		-1.005	0.201	1.004	0.322	-1.036	0.000*
cg25006073		-1.010	0.116	1.003	0.615	-1.095	0.000*
cg10553219	MMP20	-1.007	0.684	1.043	0.028*	-1.020	0.284

**Table 2e:** Methylation patterns of CpG sites on matrix metallo proteinase between Kawasaki disease patients and control subjects

Target ID	Symbol	Fold-Change	*p*-value	Fold-Change	*p*-value	Fold-Change	*p*-value
		(KD1 *vs*. HC)	(KD1 *vs*. HC)	(KD1 *vs*. FC)	(KD1 *vs*. FC)	(KD3 *vs*. KD1)	(KD3 *vs*. KD1)
cg12020179	MMP20	-1.003	0.505	1.021	0.000*	-1.047	0.000*
cg23275776		1.001	0.889	-1.005	0.247	-1.076	0.000*
cg26757793		-1.004	0.386	1.011	0.018*	-1.025	0.000*
cg02964396	MMP21	1.002	0.142	1.001	0.628	1.001	0.586
cg03437479		-1.020	0.007*	-1.006	0.359	1.011	0.120
cg03553576		-1.019	0.066	-1.011	0.276	-1.006	0.567
cg04358037		-1.009	0.006*	-1.004	0.240	-1.010	0.002*
cg05670995		-1.041	0.015*	-1.040	0.018*	-1.015	0.346
cg05721877		1.053	0.236	1.039	0.386	-1.096	0.039*
cg07954108		-1.008	0.018*	-1.012	0.001*	-1.006	0.084
cg09540803		-1.001	0.603	-1.003	0.257	-1.019	0.000*
cg11240212		-1.001	0.336	-1.003	0.035*	1.003	0.088
cg11254426		-1.011	0.044*	1.001	0.861	-1.017	0.003*
cg12666819		-1.004	0.100	-1.003	0.313	1.002	0.395
cg12854248		-1.015	0.000*	-1.008	0.010*	1.000	0.960
cg24307114		-1.011	0.001*	-1.013	0.000*	1.019	0.000*
cg24383498		-1.001	0.825	1.004	0.552	-1.023	0.000*
cg25400200		-1.013	0.044*	-1.012	0.060	-1.026	0.000*
cg25906360		-1.013	0.059	-1.006	0.349	-1.032	0.000*
cg26907302		-1.009	0.005*	-1.003	0.388	-1.007	0.030*
cg00262621	MMP23A	1.021	0.002*	1.010	0.130	-1.031	0.000*
cg00297832		1.029	0.005*	1.028	0.007*	-1.003	0.729
cg00341415		-1.014	0.012*	-1.004	0.435	-1.016	0.005*
cg00369151		1.004	0.807	-1.005	0.769	-1.025	0.179
cg01154903		-1.009	0.224	-1.007	0.332	-1.017	0.028*
cg02519897		1.002	0.705	1.001	0.818	1.018	0.003*
cg02599583		-1.006	0.762	1.022	0.267	-1.044	0.035*
cg03001942		-1.011	0.020*	-1.006	0.182	1.008	0.092
cg03136646		-1.029	0.000*	-1.018	0.001*	1.021	0.000*
cg03427058		1.011	0.512	1.051	0.003*	-1.083	0.000*
cg03930970		-1.005	0.076	1.000	0.892	-1.002	0.387
cg04437922		-1.011	0.102	1.001	0.864	-1.054	0.000*
cg04882394		-1.000	0.986	-1.012	0.000*	1.014	0.000*
cg05642789		1.011	0.561	1.060	0.003*	-1.072	0.000*
cg06084034		-1.107	0.012*	-1.040	0.318	1.019	0.639
cg06750152		-1.009	0.024*	1.007	0.073	-1.012	0.006*
cg09937190		-1.105	0.001*	-1.066	0.036*	1.038	0.217
cg11648594		1.019	0.007*	1.015	0.032*	-1.028	0.000*
cg12534645		1.025	0.042*	1.017	0.161	1.016	0.191
cg17402325		-1.016	0.009*	-1.017	0.005*	1.008	0.186

**Table 2f:** Methylation patterns of CpG sites on matrix metallo proteinase between Kawasaki disease patients and control subjects

Target ID	Symbol	Fold-Change	*p*-value	Fold-Change	*p*-value	Fold-Change	*p*-value
		(KD1 *vs*. HC)	(KD1 *vs*. HC)	(KD1 *vs*. FC)	(KD1 *vs*. FC)	(KD3 *vs*. KD1)	(KD3 *vs*. KD1)
cg18852096	MMP23A	-1.010	0.475	1.003	0.840	-1.051	0.000*
cg21265770		-1.018	0.027*	1.002	0.812	-1.034	0.000*
cg23125449		-1.009	0.013*	-1.003	0.464	1.011	0.006*
cg24688574		-1.001	0.724	1.005	0.140	-1.032	0.000*
cg27474382		1.013	0.219	1.017	0.113	-1.011	0.277
cg00262621	MMP23B	1.021	0.002*	1.010	0.130	-1.031	0.000*
cg00297832		1.029	0.005*	1.028	0.007*	-1.003	0.729
cg00369151		1.004	0.807	-1.005	0.769	-1.025	0.179
cg01154903		-1.009	0.224	-1.007	0.332	-1.017	0.028*
cg02599583		-1.006	0.762	1.022	0.267	-1.044	0.035*
cg03001942		-1.011	0.020*	-1.006	0.182	1.008	0.092
cg03136646		-1.029	0.000*	-1.018	0.001*	1.021	0.000*
cg03427058		1.011	0.512	1.051	0.003*	-1.083	0.000*
cg03930970		-1.005	0.076	1.000	0.892	-1.002	0.387
cg04854189		-1.008	0.030*	-1.003	0.348	-1.020	0.000*
cg04882394		-1.000	0.986	-1.012	0.000*	1.014	0.000*
cg05642789		1.011	0.561	1.060	0.003*	-1.072	0.000*
cg06080300		-1.068	0.000*	-1.043	0.000*	1.026	0.010*
cg06084034		-1.107	0.012*	-1.040	0.318	1.019	0.639
cg06912282		-1.115	0.000*	-1.059	0.000*	1.076	0.000*
cg09937190		-1.105	0.001*	-1.066	0.036*	1.038	0.217
cg11648594		1.019	0.007*	1.015	0.032*	-1.028	0.000*
cg12534645		1.025	0.042*	1.017	0.161	1.016	0.191
cg17402325		-1.016	0.009*	-1.017	0.005*	1.008	0.186
cg18852096		-1.010	0.475	1.003	0.840	-1.051	0.000*
cg23125449		-1.009	0.013*	-1.003	0.464	1.011	0.006*
cg02288791	MMP24	-1.005	0.280	1.003	0.421	-1.035	0.000*
cg03260792		1.000	0.806	-1.002	0.252	1.001	0.522
cg04316754		-1.013	0.056	1.001	0.832	1.046	0.000*
cg12483876		-1.003	0.676	-1.007	0.289	1.056	0.000*
cg15270813		-1.017	0.088	-1.005	0.610	1.039	0.000*
cg00456894	MMP25	1.000	0.838	1.004	0.000*	1.000	0.716
cg01270736		-1.021	0.002*	-1.026	0.000*	1.005	0.448
cg02616220		-1.005	0.231	1.011	0.008*	-1.048	0.000*
cg02988422		-1.014	0.123	-1.013	0.152	1.009	0.325
cg05168200		1.004	0.330	1.020	0.000*	-1.030	0.000*
cg06697694		1.003	0.172	1.002	0.264	1.020	0.000*
cg08607821		-1.008	0.095	-1.005	0.280	-1.005	0.285
cg10620273		1.001	0.654	1.007	0.001*	-1.004	0.040*
cg16856286		-1.002	0.437	-1.004	0.211	1.008	0.016*

**Table 2g:** Methylation patterns of CpG sites on matrix metallo proteinase between Kawasaki disease patients and control subjects

Target ID	Symbol	Fold-Change	*p*-value	Fold-Change	*p*-value	Fold-Change	*p*-value
		(KD1 *vs*. HC)	(KD1 *vs*. HC)	(KD1 *vs*. FC)	(KD1 *vs*. FC)	(KD3 *vs*. KD1)	(KD3 *vs*. KD1)
cg19612376	MMP25	-1.004	0.237	1.001	0.789	-1.003	0.295
cg22053861		-1.004	0.061	-1.002	0.255	-1.001	0.490
cg23531828		1.002	0.867	-1.012	0.181	-1.014	0.125
cg25584355		1.001	0.585	-1.005	0.008*	1.001	0.496
cg26705553		-1.006	0.001*	1.009	0.000*	-1.003	0.172
cg26942943		-1.006	0.006*	-1.002	0.444	1.010	0.000*
cg02706104	MMP26	-1.005	0.147	1.000	0.990	-1.038	0.000*
cg05316601		-1.013	0.000*	-1.003	0.334	-1.030	0.000*
cg09805271		-1.013	0.176	-1.009	0.335	-1.104	0.000*
cg12493906		-1.028	0.000*	-1.008	0.143	1.017	0.002*
cg17992177		-1.018	0.001*	-1.006	0.261	-1.032	0.000*
cg23389785		-1.009	0.015*	-1.006	0.108	1.000	0.943
cg06259570	MMP27	-1.012	0.012*	-1.011	0.019*	-1.037	0.000*
cg09758894		-1.011	0.015*	1.008	0.082	-1.015	0.001*
cg13252516		1.003	0.329	1.006	0.080	-1.038	0.000*
cg18932968		-1.003	0.302	1.013	0.000*	-1.025	0.000*
cg25277638		-1.024	0.039*	-1.009	0.456	1.004	0.730
cg01134296	MMP28	-1.024	0.000*	-1.019	0.000*	1.020	0.000*
cg01352921		-1.001	0.304	-1.002	0.201	1.004	0.001*
cg01622018		-1.062	0.000*	-1.022	0.046*	1.018	0.091
cg04790749		-1.004	0.614	1.009	0.273	-1.063	0.000*
cg07330075		-1.005	0.184	1.009	0.021*	-1.029	0.000*
cg07546558		1.009	0.002*	1.008	0.003*	-1.011	0.000*
cg07916589		-1.005	0.231	1.006	0.164	1.004	0.378
cg09357580		1.004	0.004*	1.003	0.031*	-1.003	0.018*
cg09730211		1.003	0.143	1.007	0.003*	-1.014	0.000*
cg09807148		-1.014	0.000*	-1.002	0.444	-1.002	0.501
cg12700449		-1.028	0.000*	-1.017	0.001*	-1.001	0.893
cg13326172		1.001	0.460	-1.005	0.001*	1.005	0.001*
cg16246294		1.002	0.486	1.014	0.000*	-1.015	0.000*
cg25172682		1.018	0.095	1.009	0.415	-1.042	0.000*

For simplicity, we listed the p values only to 10-3. Actually, the CpG marker cg10505873 (the target for further examination) has p value < 10-5 among all of the three comparisons (KD1 vs. FC, KD1 vs. HC and KD3 vs. KD1). When examined with the FDR (with Bonferroni correction), cg10505873 is also significant among the three comparisons.

KD1: Kawasaki disease before IVIG treatment; KD3: Kawasaki disease > 3 weeks after IVIG treatment; FC: febrile control; HC: healthy control.

### MMP-8, -9 and -25 expressions in peripheral white blood cells (WBCs) of KD patients and controls

**Figure 3:**
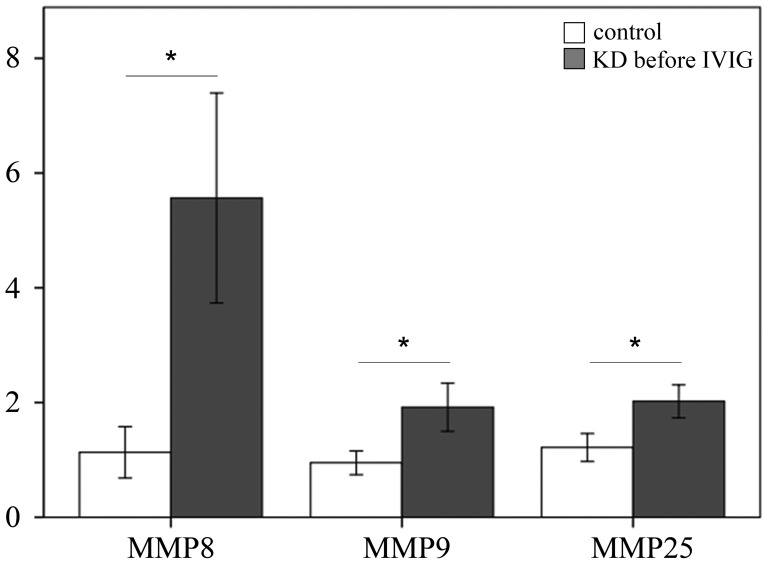
Analyses of matrix metalloproteinase (MMP) -8, -9, and -25 mRNA in the peripheral blood mononuclear cells of 31 patients with KD and 46 controls using a real-time quantitative polymerase chain reaction Data are expressed as mean ±standard error. *indicates a p < 0.05 between the groups.

**Figure 4:**
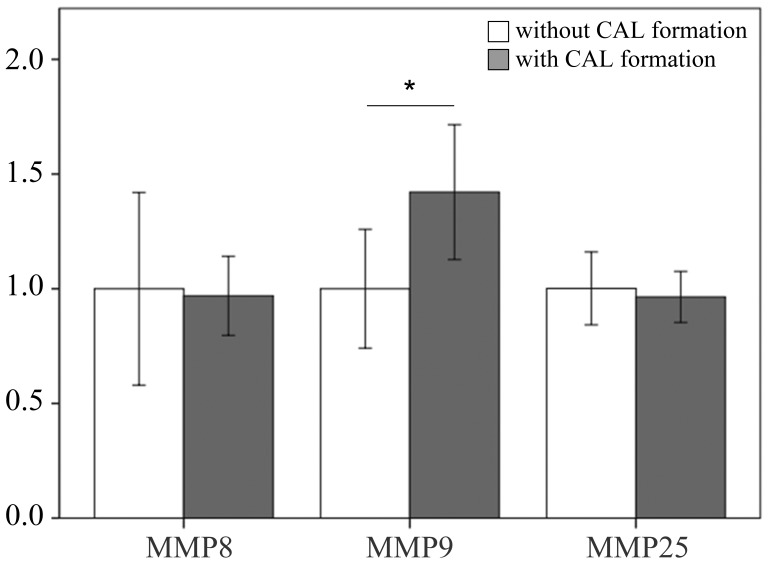
Comparison of matrix metalloproteinase 9 in patients with KD without (*n* = 22) and with (*n* = 9) coronary artery lesion before being treated with intravenous immunoglobulin Data are presented as mean ±standard error. *indicates a *p* < 0.05 between the groups.

We examined the mRNA levels of MMP-8, -9, and -25 in a separate cohort consisting of 31 KD patients and 46 controls (23 healthy and 23 febrile controls) using real-time PCR. We found higher MMP-8 (*p* =0.004), MMP-9 (*p* =0.024), and MMP-25 mRNA levels (*p* = 0.002) in the WBCs of KD patients than in those of the controls, as shown in Figure 3. Such findings are consistent with the Affymetrix GeneChip^®^ Human Transcriptome Array 2.0 results. Moreover, the mRNA level of MMP-9 was greater in KD patients who developed CAL than those who did not (*p* = 0.035) (Figure 4).

## DISCUSSION

To the best of our knowledge, ours is the first study to comprehensively survey global DNA methylation levels and transcripts of MMPs between KD patients and control subjects. Our noteworthy observations include the epigenetic hypomethylation and upregulation of MMP-9 in KD, as well as the increase of mRNA levels of MMP-9 in KD patients with CAL formation.

Cardiovascular complications are the leading cause of morbidity and mortality in KD patients [14]. Coronary dilatation followed by aneurysm occurs in 20-25% of KD children who do not receive treatment [15, 16]. Undergoing IVIG treatment can effectively decrease the likelihood of CAA formation, but its effect on etiology aneurysm formation remains unclear [14, 17]. An increasing amount of evidence has also suggested that children with KD are at a long-term risk for subsequent cardiovascular events, possibly due to the endothelial dysfunction and inflammation of the coronary and systemic arteries [18, 19]. MMPs have an important controlling influence regarding maintaining coronary vascular wall structure and function [4]. Previous studies have reported a significant correlation between MMP-9 with coronary artery disease [20] and outcomes after acute myocardial infarction [21]. The overexpression of MMP-2, -8, -9, and -12 was related to abdominal aortic aneurysm [22]. Likewise, MMP-8, -9, and -12 seem to be associated with collagen I and collagen III, as well as their degradation products and vascular remodeling [22]. More importantly, Newby *et al.* proposed that MMP-mediated macrophage invasion and tissue destruction fosters the ideal environment for atherosclerotic plaque rupture [23].

While increasing evidence has indicated that cytokine profiles are related to the pathogenesis of KD, the actual CALs involved remain unclear. MMP-3 and -12 haplotypes have been correlated with CAA formation in patients with KD [7], while MMP-11 polymorphism may be correlated with KD in Korean children [24]. Furthermore, diffuse and strong staining of MMP-9 was observed in coronary lesions of children who died from KD [25], as was an increased MMP-9 expression in vascular endothelial cells induced by the serum of KD patients [26]. Remarkably, MMP-9 activity can aid in breaking down elastin [27], so hampering such activity can improve coronary artery inflammation in a KD animal model [28]. IVIG treatment can also inhibit monocytes that express MMP-9, thus decreasing chemotactic migration of monocytes and CAL development [29]. was Another study showed that treatment with TNF-α [30] and advanced glycation end products decrease the promoter DNA methylation and increase the endogenous expression of MMP-9 in the human keratinocyte cell line [31]. Moreover, oxidized low-density lipoprotein significantly increased primary human aortic smooth muscle cell migration by decreasing DNA methylation levels and increasing MMP-9 expression [32], which supports our finding that expression abundance of MMP-9 can be altered and negatively correlated with the methylation profile. In our previous studies, we have described KD patients showing remarkably increased mRNA expression in TLRs and hypomethylation at TLR gene promoters [13]. Interestingly, Soria-Valles at al. showed that MMP-25 could activate the innate immune response [33]. Similarly, our study has also found increased transcripts of MMP-8, MMP-9, and MMP-25 in KD, indicating that these proteinases may be potential therapeutic targets for KD.

Prostaglandin E2 (PGE2) is a prostanoid created by almost all vascular cell types and takes part in vascular wall remodeling by regulating MMP activities [34]. PGE2 can further stimulate MMP-9 expression in inflammatory cells [35], and we discovered that plasma PGE2 is correlated with the prevention of IVIG resistance and CAL formation through CD40L in KD [36]. A previous study found that transcripts correlated with MMP-9 and interferon-gamma in PBMC in patients with coronary artery ectasia [37], while we observed an association among IFNG gene polymorphisms, susceptibility to KD, IVIG responsiveness, and plasma IFN-γ levels in KD patients [38]. In this study, we demonstrated that MMP-9 was not only upregulated in KD but also had a significant correlation with CAL formation in Taiwanese KD patients. These observations provide insight into the association among inflammatory cytokines, matrix metalloproteinases, and CAL formation in KD. As a result, we may use MMP gene expression and DNA methylation as KD biomarkers to develop an effective KD diagnosis model.

This study has various limitations. First, we do not know if potential changes in methylation occur due to an association with decreased inflammation or due to the IVIG treatment. Second, we use total WBCs in this study, but determining whether specific types of blood cells, such as regulatory T cells, or specific markers show epigenetic changes with IVIG or KD would be productive and interesting.

## CONCLUSIONS

Our report is the first to observe DNA hypomethylation, an increased MMP-9 transcript in KD compared to controls and an upregulation of MMP-9 in KD patients with CAL formation.

## MATERIALS AND METHODS

### Patients

For this study, we recruited a total of 54 subjects from Kaohsiung Chang Gung Memorial Children’s Hospital in Taiwan in this study. Of those, 18 subjects were fever controls (with fever but not diagnosed to have KD), 18 subjects were healthy controls (with a history of neither fever nor KD), and the remaining 18 subjects were KD patients who had had samples collected both before and after IVIG treatment, met the American Heart Association diagnosis criteria, which is characterized by prolonged fever for more than five days, conjunctivitis, diffuse mucosal inflammation, polymorphous skin rashes, indurative edema of the hands and feet associated with peeling of the finger tips, and non-suppurative lymphadenopathy [39, 40], and received IVIG treatment at the hospital. Using a separate cohort, we performed real-time quantitative PCR validations of mRNA levels in 31 KD patients, 23 healthy controls, and 23 febrile controls. The patients in the fever control group had diagnoses of acute tonsillitis, croup, acute bronchitis, or urinary tract infection. We took peripheral blood samples from KD patients at two points: prior to being treated with IVIG (pre-IVIG) and at least three weeks after completing IVIG treatment (37.8 ± 4.0 days), as described in our previous studies [36]. A CAL was defined as a coronary artery with an internal diameter of at least 3 mm (4 mm if the subject was older than 5 years) or a segment with an internal diameter at least 1.5 times larger than that of an adjacent segment, as observed through echocardiography [14, 41]. This study received approval from the Chang Gung Memorial Hospital’s Institutional Review Board (IRB No.:101-4618A3), and we obtained written informed consent from the parents or guardians of all subjects. All of the methods used comply with the relevant guidelines established.

### Experiment design

First, we collected whole blood samples from the subjects and subjected said samples to WBC enrichment, as described in a previous study [12]. The enriched WBC samples were then subjected to either RNA or DNA extraction. We used an isolation kit (mirVana™ miRNA Isolation Kit, Catalog number: AM1560, Life Technologies, Carlsbad, CA) following the manufacturer’s instructions to isolate total RNA and measured the collected RNA samples using Bioanalyzer (ABI) and Qubit (Thermo) to calculate both the quality (RIN value) and quantity of the RNA. All RNA samples passed the criterion of RIN≧7. We isolated the DNA samples and treated them with bisulfite as previously described in another study [42].

### Gene expression profiling with microarray

For strong, unbiased results, pooled RNA libraries were created by evenly pooling six RNA samples, which resulted in three pooled normal control, three fever control, three pre-IVIG, and three post-IVIG libraries. We submitted the pooled RNA samples to microarray assay to establish the gene expression profiles. We further performed profiling with GeneChip^®^ Human Transcriptome Array 2.0 (HTA 2.0, Affymetrix, Santa Clara). We used the WT PLUS Reagent kit to prepare the RNA samples and then performed hybridization on the HTA 2.0 microarray chips. In accordance with the Affymetrix instruction manual, the raw data of the HTA 2.0 chips underwent quality control examination, as previously described [13].

### DNA methylation profiling with Illumina M450K BeadChip

We used Illumina HumanMethylation450 (M450K) BeadChip to perform genome-wide screening of DNA methylation patterns. The M450K BeadChip program was created to detect methylation patterns of approximately 450,000 CpG markers, thus spanning the entire human genome. Additional information about M450 BeadChip can be found at the following website: http://support.illumina.com/array/array_kits/infinium_humanmethylation450_beadchip_kit.html. For each M450K BeadChip assay, we applied 200 ng of bisulfite-converted genomic DNA in accordance with the manufacturer’s instructions, as previously described [12]. Then, we calculated the methylation percentage of cytosine for each CpG marker in each sample, referring to them as β values.

### RNA isolation and real-time quantitative RT-PCR

In order to quantify the mRNA levels of MMP-8, -9, and -25, we performed real-time quantitative PCR using the ABI 7700 Sequence Detection System (TaqMan; Applied Biosystems, Inc., Foster City, CA). We separated the total mRNA from the WBC using an isolation kit (mirVana™ miRNA Isolation Kit, Catalog number: AM1560, Life Technologies, Carlsbad, CA), following the manufacturer’s instructions. We performed PCR using a SYBR Green PCR Master Mix containing 10 μM of specific forward and reverse primers. The relative quantification of gene expression was carried out based on the comparative threshold cycle (C_T_) method, through which the target amount was determined to be 2^−(ΔCT target − Δ CT calibrator)^ or 2^−ΔΔCT^ [43]. Primers were designed to amplify the target genes, which are provided in Table 4. We performed all experiments twice for verification and to validate the amplification efficiencies.

**Table 3:** Basal characteristics of controls and patients with KD

Characteristic	Healthy controls	Febrile controls	Patients with KD
	(*n* = 41)	(*n* = 41)	(*n* = 49)
Male gender, n (%)	26(63.4)	25(60.9)	34(69.3)
Mean (± SD), age (y)	6.1 ± 4.7	3.3 ± 2.6	1.8 ± 1.7
CAL formation			18(36.7%)

CAL, coronary artery lesion; KD, Kawasaki disease; SD, standard deviation

**Table 4:** Primers were designed to amplify target genes

Gene symbol	Accession number	Hybridization	Primers (5’ to 3’)	Primer location	Amplicon size (bp)
RNA18S5	NR_003286.2	forward	GTAACCCGTTGAACCCCATT	1577 - 1597	151
		reverse	CCATCCAATCGGTAGTAGCG	1708 - 1727	
MMP8	NM_002424	forward	TTGGGTTGAATGTGACGG	308 – 326	156
		reverse	GTATAGTTTCGAATCCTGTAGGT	439 - 463	
MMP9	NM_004994	forward	TCCAACCACCACCACAC	1378 – 1396	178
		reverse	CGGACTCAAAGGCACAGTA	1534 – 1555	
MMP25	NM_022468	forward	GGACACTCACTTTGACGAT	852 – 873	192
		reverse	GAGACAGGCGGTACTTG	1026 - 1043	

### Statistical analysis

We have presented all data as mean ± standard error. The chips that passed the quality control criteria were analyzed with Partek (Partek, St. Louis), commercial software designed specifically to analyze microarray data. Using Partek, we conducted ANOVA analysis and reported the p-values of the comparisons of interest as previously described [13].

We applied Student’s t-test or one-way ANOVA, as appropriate, to analyze the quantitative data. Furthermore, we evaluated any data changes before and after IVIG treatment with the paired sample *t-*test. All statistical analyses were carried out using SPSS version 22.0 for Windows XP (SPSS, Inc., Chicago, USA), and a two-sided p-value less than 0.05 was considered statistically significant.
